# FieldML: concepts and implementation

**DOI:** 10.1098/rsta.2009.0025

**Published:** 2009-05-28

**Authors:** G. Richard Christie, Poul M.F. Nielsen, Shane A. Blackett, Chris P. Bradley, Peter J. Hunter

**Affiliations:** 1 Auckland Bioengineering Institute, University of AucklandAuckland 1142, New Zealand; 2 Spark Dental Technology LtdNorth Shore City 0632, New Zealand; 3 Department of Physiology, Anatomy and Genetics, University of OxfordOxford OX1 2JD, UK

**Keywords:** field, modelling, computation, serialization, FieldML

## Abstract

The field modelling language FieldML is being developed as a standard for modelling and interchanging field descriptions in software, suitable for a wide range of computation techniques. It comprises a rich set of operators for defining generalized fields as functions of other fields, starting with basic *domain fields* including sets of discrete objects and coordinate systems. It is extensible by adding new operators and by their arbitrary combination in expressions, making it well suited for describing the inherent complexity of biological materials and organ systems. This paper describes the concepts behind FieldML, including a simple example of a spatially varying finite-element field. It outlines current implementations in established, open source computation and visualization software, both drawing on decades of bioengineering modelling software development experience.

## 1. Introduction

A field is an abstraction of some quantity defined over a domain. To mathematically *model* the physics of real-world bodies, fields describing physical state and other properties at locations within the domain are approximated by functions based on a finite set of parameter values.

The combinations of functions able to describe a field to ever greater accuracy are without limits. However, this is balanced by a need to reduce the number and complexity of functions and the corresponding size of parameter sets to minimize storage and computation overhead. Complexity is frequently reduced by assuming some degree of continuity in the representation of field values and derivatives, but this must be physically justifiable. Such assumptions may be reasonable at one scale but not at another: the deformation of muscle tissue appears continuous at the macroscopic tissue scale but this may only be the bulk behaviour of more detailed mechanisms at the scale of individual sarcomeres and interconnecting collagen fibres. To support the widest gamut of problems, field formats must not mandate the assumptions of continuity and the other aspects of representation.

The field modelling language FieldML is being developed to represent fields with few restrictions on function, domain type and dimension. FieldML intends to address the requirements for representing fields for common modelling techniques including finite elements, boundary elements, finite difference and finite volumes, but also more general fields unrestricted by the current solution techniques.

FieldML avoids complex data structures for defining fields such as meshes with fixed notions of connectivity, and instead defines fields in terms of more abstract domains including sets and coordinate systems. These domains are each a type of field whose values are object references or coordinate system locations. The majority of fields are defined by mathematical operators or the result of algorithms acting on values of other fields, whether they are prescribed values of *domain fields* or computed from them. FieldML is expressive since the value of each field at domain locations is explicitly stated by functions. It is also extensible via the definition of new field operators or types, and their combination in expressions. It supports software reuse because each field operator need only know how to deal with the type of values of the fields it operates on, irrespective of the ultimate domain they are defined over. FieldML eliminates the need to have separate data formats for *piecewise* continuous fields, tabulated parameters and discrete variables.

Although FieldML is primarily intended to describe the structure and parametrization of spatial fields, its rich set of field operators including the spatial and temporal operators of differential and integral calculus can in combination also describe the physical principles that govern biological behaviour such as reaction–diffusion processes, large-deformation elasticity theory or computational fluid dynamics. For example, an elastic strain energy function can be considered a field and expressed using FieldML. However, the solution or minimization of these equations is not a part of FieldML.

FieldML is being developed as:

- a data model defining concepts and basic objects from which fields are constructed,
- open source software implementations and application programming interfaces (APIs) for modelling fields following the data model, and
- one or more native FieldML serialization formats and API to support interchange of field descriptions.

The primary intention of this paper is to communicate the underlying data model for FieldML, and to outline the development of software implementations for computation and visualization. Concepts are illustrated with a mock-up serialization of a spatially varying finite-element field, but the issue of developing FieldML serialization formats is mostly left to later work and is reliant on consensus on the data model, and the availability of software interfaces and implementations. Also discussed is the overlap with the CellML markup language (www.cellml.org), which has similar capabilities for describing mathematical relationships between variables, but lacks spatial variation.

Many of the ideas presented here have already been successfully implemented in software, but other areas are still at the proposal stage. The authors invite feedback and other contributions towards the development of the FieldML standard.

## 2. Background

A significant influence on FieldML has been the CMISS modelling software (www.cmiss.org), which supports complicated representations of finite-element fields using basis functions with high-order continuity and flexible parameter mappings. CMISS models use fields to express most simulation variables, including geometry, material properties as well as dependent variables. It is able to use different basis functions for each field component defined over the same topology. This contrasts with conventional finite-element representations, which restrict fields to using just a few simple element types, mixing function with topology. In addition, they often treat geometry and material properties as special cases, distinct from other fields. Examples include the General Mesh Viewer format (http://www-xdiv.lanl.gov/XCM/gmv) and EXODUS II format (http://endo.sandia.gov/SEACAS/Documentation/exodusII.pdf). The authors consider these formats to be limiting for many of the problems being encountered in bioengineering. Christie *et al*. (2002) showed the benefits of defining fields by mathematical operations on other fields including cases where the fields have a nonlinear relationship to field parameters, as illustrated later by the muscle fibres in figure 7. It is noted that the more varied field representations in CMISS come at the cost of greater software complexity, which FieldML intends to reduce by replacing fixed-functionality codes with modular combinations of basic field operations.

The libMesh project (Kirk *et al*. 2006, http://libmesh.sourceforge.net) is noted as a modern framework for the numerical simulation of partial differential equations, which supports distributed parallel systems and adaptive mesh refinement. To model various physics problems, it is able to describe an arbitrary number of spatially varying fields (here termed ‘variables’), which is a requirement common with FieldML.

Like FieldML, the Sets and Fields (SAF) modelling system (see Miller *et al*. 2001) concludes that it is beneficial to construct complicated field representations out of a few reusable building blocks, namely sets, relations and fields. Despite its generality, SAF has been able to replace more restrictive data formats for model storage and interchange while maintaining a high performance. FieldML packages its basic concepts differently, considering sets as a specialized domain field type, while relations are either a field operator type or in some cases expected to be communicated from a subsequent metadata specification. A further difference is that parameters are also a field type in FieldML. In contrast to SAF, FieldML is being developed as a basis for modelling software, not just for serialization. Further discussion on combining efforts with SAF appear worth while if SAF continues to be actively developed under appropriate licensing terms.

Sandia National Laboratories' SIERRA Framework (Edwards 2006, http://csmr.ca.sandia.gov/projects/ftalg/Edwards02.pdf) is a further effort to find common abstractions for modelling data structures. The project aims to unify core data structures and facilities in several computation codes, reduce maintenance costs, enhance performance across the suite of codes and facilitate interoperability to support massively parallel multi-physics simulations.

## 3. FieldML concepts

### (a) Field

A field is commonly defined as some values varying over a spatial domain. For computation, a field is often implemented as a function mapping the domain locations over which it is defined to the field values. FieldML generalizes these definitions in a few areas, which are as follows:

- The domain of a field need not be spatial in the literal sense; space could alternatively represent time or any other solution variable.
- Domains need not be continuous or connected spaces; hence valid domains include sets of discrete objects and whole models considered as a single unit.
- FieldML fields are considered as functions, but this is extended to domains themselves whose values are prescribed rather than computed, in effect implementing the identity function.
- Field values are unlimited in type and may represent integers, strings, object references and structures in addition to the usual scalar, vector and tensor real numbers. Field functions ‘return’ values of a certain type.

True to the common definition, a FieldML field represents a family of related values over some domain, but in software terms it is a mechanism to return values at prescribed locations in its domain, be that space, time, continuous, discrete or other.

### (b) Field type

An important step towards the current thinking on FieldML was the idea of deriving fields by simple mathematical operations on other fields. The result of a field operator is another field; one could therefore consider each field to be an instance of an operator acting on one or more input fields, so each type of field operator could alternatively be considered as a ‘field type’.

This leads to a convenient implementation in object-oriented languages: the field type is an abstract base class with pure virtual methods for evaluating its values at domain locations, querying value types and other generic tasks. Actual fields are created as instances of derived classes, which implement particular mathematical operators acting on the values of other fields and objects. Even complicated field representations such as a finite-element interpolation over a mesh can be reduced to basic operators combining basis functions defined as mathematical expressions of coordinate chart locations, and various operators for extracting element parameters from fields defined over discrete ‘node’ objects.

Parameters are considered a special field type whose values are stored rather than calculated. The ability to substitute a fixed parameter field with a computed field is a powerful tool for constructing complex field representations.

Domain objects such as meshes and coordinate systems can also be treated as fields at an abstract level. The range of permissible values of domain fields forms a part of their definition. Values indicating locations in the domain must be specified to evaluate other fields depending on them. Considering domain objects as fields at an abstract level does not lead us to a dilution of their concepts. In many cases, special field operators will be needed to work with particular domain types, such as a piecewise function defined over a mesh domain. Each field type, including domain fields, may have additional functionality (exposed via extra software interfaces) as needed to fulfil its role. Software can evaluate fields without knowing the details of the operations being performed, but needs to know intimately how to work with domains, for example to iterate over elements and integrate or visualize fields over element coordinate systems. Note that the mathematical notion of the ‘domain of a field’ could be the range of another field, so one has to be careful with terminology.

**Figure 1:**
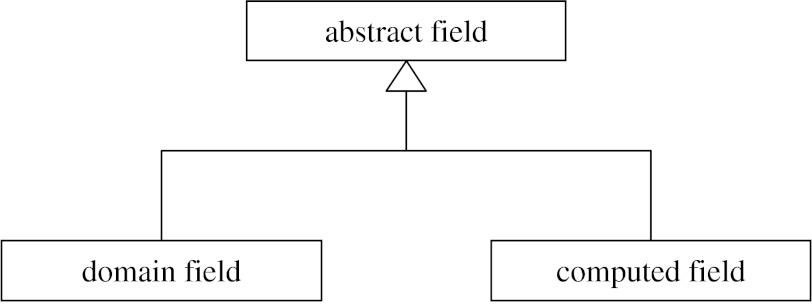
Class diagram showing main categories of FieldML field types.

Figure 1 shows a skeleton class diagram for FieldML field types. The base abstract field presents all common features of fields including the type of its values, methods to determine which domain field it ultimately depends on and methods for evaluating the field, given values of fields it depends on. The main division below this point is into domain fields, owing to their special role at the source of a chain of evaluation, and the remaining fields whose values are computed, including stored value parameter fields.

The unified treatment of fields, parameters and domain objects permits the consistent use of their values, and simplified specification of operators such as derivatives, which can be evaluated with respect to fields or their components, parameters and domain locations.

FieldML field types implement the most basic non-reducible operations to support maximum reuse. The simplest field types to implement are common mathematical operations on numerical-valued fields including add, subtract, multiply, divide, trigonometric functions, vector functions, matrix functions and more.

Some of the main field operators and types proposed are as follows.

- *Ensemble*. A domain field consisting of a collection of objects treated as a whole, which may represent a set of nodes, elements, particles or other objects. This can be extended to allow ensembles of ensembles to support the types of hierarchical domains described in §3*f*.
- *Coordinate system*. Declaration of an *n*-dimensional continuous domain, possibly restricted to a subset of *R*^*n*^. Section 3*c* describes several coordinate systems and their combination with ensembles to define meshes.
- *Piecewise*. A field implemented by one or more operators defined over all or parts of an *ensemble* domain. A common use is to define interpolation using basis functions over a set of elements. Several piecewise fields are used in the example in §4.
- *Parameter*. Stores literal field values per ensemble object it is defined over.
- *Import*. Imports a field from the same or other FieldML model but substituting zero or more of its source fields with local fields. This is one of the few ways in which fields from other models can be reused. It is important to control inter-model dependences to make it clear what information needs to be serialized with a model, and to handle propagation of change messages from other models in software implementations.
- *Derivative*. Calculates derivatives of fields with respect to other continuous real-valued scalar or vector fields. Other derivative field types include vector field divergence, gradient and curl operators.
- *Function inverse and compose*. Inverting a coordinate field to return a location in its domain from coordinate values, then evaluating another field at that location, can make a field effectively a function of another field as if it were its domain. Practical uses include implementing time-dependent fields, arbitrary texture coordinates in image-based fields and general embedding.
- *Logical and conditional fields*. These enable field values to depend on Boolean expressions giving custom control of field values.
- *Image and image processing fields*. These fit elegantly into the field abstraction, enabling integration with piecewise or finite-element representations.

### (c) Domain fields

A field definition is incomplete without the specification of the domain over which it is defined, and whose values must be prescribed in order to evaluate the field. FieldML domains are principally divided into sets of objects (ensembles) and continuous coordinate systems or element *charts*, or their combination into piecewise coordinate systems, referred to as an *atlas* or *mesh*.

**Figure 2:**
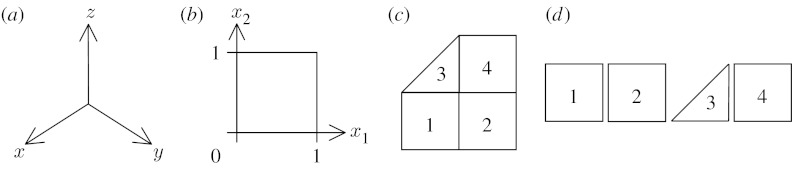
Continuous domains: (*a*) coordinate system chart covering *R*^3^; (*b*) chart restricted to [0,1] in two dimensions; (*c*) mesh or atlas, a set of element charts; and (*d*) mesh without connectivity are shown.

Figure 2 illustrates several continuous and piecewise continuous domains.

Figure 2*a* shows a three-dimensional coordinate system covering all of *R*^3^, while figure 2*b* shows a coordinate system restricted to part of *R*^2^. Each of these domains is alternatively referred to as *chart*; locations within them are specified by a number of coordinates equal to their dimension. Numerical problems over complex geometries are usually solved using a *mesh* or *atlas* domain as shown in figure 2*c*, which consists of a set of *elements* each with its own coordinate chart. This is due to the ease with which the domain can be mapped by a set of charts of simple shape, but also to support piecewise field functions.

Figure 2*d* shows that, without additional information, each element chart is independent and unconnected. A FieldML mesh principally provides an unambiguous coordinate system for identifying points in the domain. It is not seen as a requirement that the domain objects (elements) maintain custom information about inter-element connectivity, but rather that this be conveyed by fields defined over these domain objects. Connectivity can be inferred for a field from shared parameters and functions along element boundaries. Such mappings can be changed during a simulation or be made a function of another field such as the time domain. If it becomes necessary to communicate cached mesh connectivity information in a more convenient format, this can be done via the general return values allowed by fields, discussed in §3*d*. An example is a field whose values are a list of shared global nodes (the element's ‘local node list’—often already part of a finite-element field definition) or references to matching face elements for each element in a mesh. These generalized fields offer advantages over hard-coded members of element objects in traditional modelling codes in that connectivity can be described in multiple ways; it can be dynamically calculated or omitted if not needed.

Ensemble fields without associated element charts are used to represent sets of zero-dimensional objects such as nodes, particles, data points and other entities. The example of §4 has a node ensemble with a parameter field defined over it, supplying nodal coordinates for element interpolation.

Section 3*e* introduces the model object, which is a hierarchical container for FieldML fields. Some fields consider the whole model as a point domain for the definition of fields that are invariant across any subdomains making up the model. These fields may represent model-wide constants or simple variables; they are intended to allow FieldML to work as easily with lumped-parameter models as with models requiring complex spatial and temporal variation.

### (d) Field values

Values of FieldML fields include real and complex scalar, vector, matrix/tensor quantities, manipulated in software as floating-point numeric types. The parameters from which fields are calculated are often stored at lower precision to reduce storage requirements, and are sometimes not floating-point values: integer values are common for image-based fields. Locations in domains consist of references to discrete objects or, in the case of meshes, a combination of element reference and element chart coordinates. Allowing fields to return domain locations in other meshes and evaluating fields defined there enables powerful concepts such as embedding and supports familiar constructs such as element local node lists used in finite-element fields. These non-standard field values are the mechanism by which FieldML avoids the use of hard-wired data structures for defining fields, which are generally found to limit possible field representations.

Field values are incomplete without additional attributes and metadata to aid interpretation. This includes units for each field or field component, which FieldML implementations will use to prevent operators combining incompatible fields, and also to establish units of derived fields. Other important attributes include whether values are to be interpreted as purely real numbers, complex pairs, quaternion or other; whether components of vectors and tensors are covariant, contravariant or mixed with respect to a basis set; and coordinate systems with respect to which geometric fields are defined. Each of these has a bearing on how certain field operators work. A proposal for working with coordinate systems is presented in the example later, but other attributes are not discussed further in this paper.

### (e) FieldML models

At the highest level, FieldML defines the *model* object, which is a hierarchical container of fields and submodels. Models (termed ‘regions’ in the software implementations of §5) enable encapsulation, separating field namespaces so that multiple models and submodels may coexist without interference.

**Figure 3:**
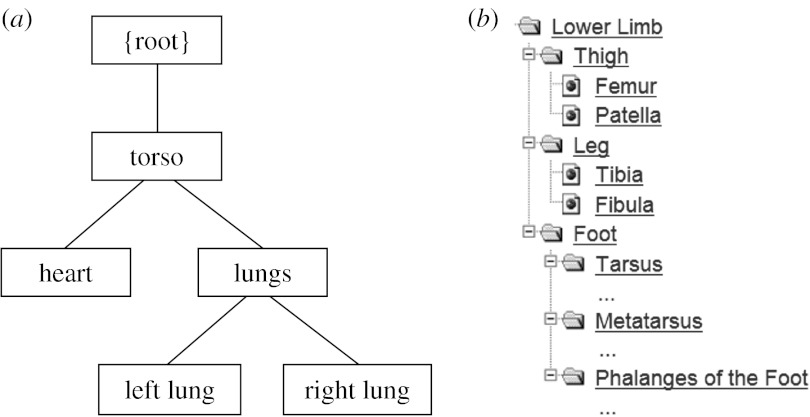
Example model hierarchies from bioengineering: (*a*) composite model of heart and lungs and (*b*) hierarchy of skeleton lower limb.

Modellers and modelling software may assign whatever meaning to each model object as deemed appropriate. FieldML models may correspond to parts and assemblies in computer-aided design data. In the modelling of organs and other biological systems, it may be practicable to use a single model to represent a whole organ or cell, or alternatively encapsulate distinct parts into submodels. The musculoskeletal system fits well into model hierarchies matching traditional anatomical classifications. Two examples of model hierarchies are given in figure 3.

The Cmgui application (see §5) attaches a graphical rendition to each model/region in order to visualize its fields. In a similar manner, computation codes may associate solution matrices and other data with FieldML models.

### (f) Hierarchical meshes and fields

Two hierarchical mesh concepts are on the FieldML roadmap and are described here to give an indication of the type of field representations FieldML intends to be able to support.

**Figure 4:**
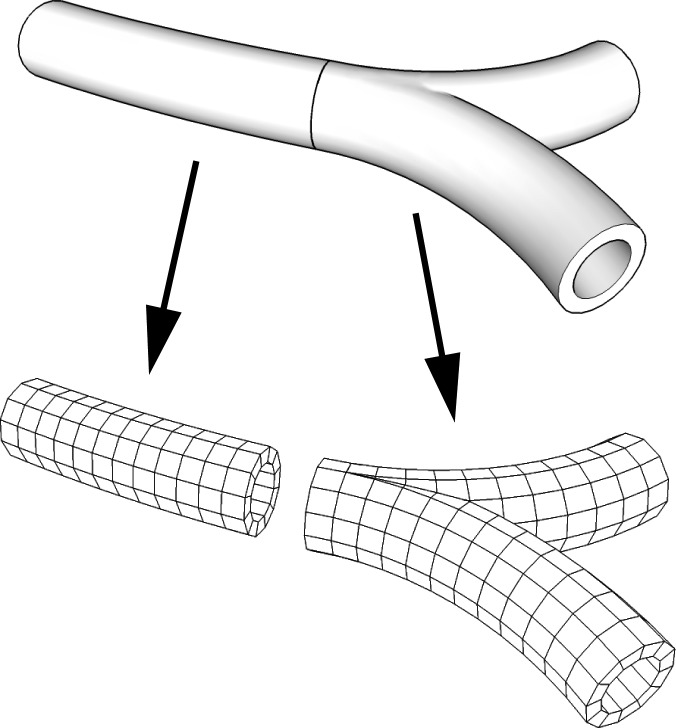
Hierarchical model construction by aggregation of template meshes and fields.

The first is the construction of complex models out of template meshes and fields as illustrated in figure 4.

Here, discretized models of a simple tube and a bifurcating tube section are combined into an aggregate model, reusing mesh and field definitions in each part. The model maintains a tree of constituent meshes, and couples degrees of freedom on the common boundary.

**Figure 5:**
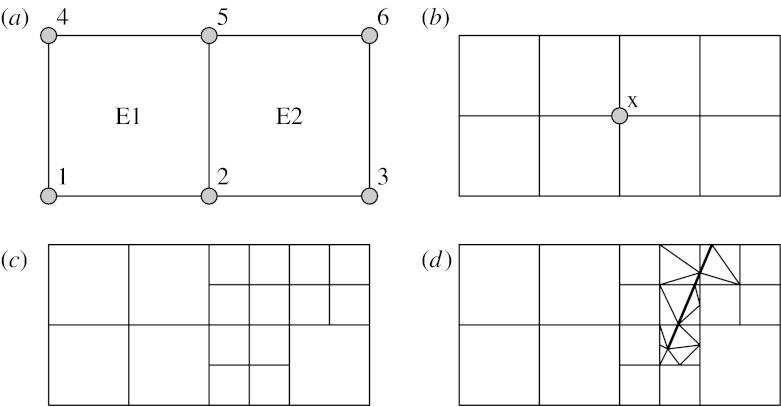
(*a–d*) Adaptive refinement of a mesh to represent detail of a cut.

The second hierarchical meshing concept is adaptive refinement, where the density of piecewise functions making up a field is increased to approach a solution to the desired accuracy. Figure 5*a–c* illustrates regular refinement of an initially two-element mesh in selected elements while figure 5*d* illustrates irregular refinement by triangles to fit the line of a cut to the body.

Assuming the field in each element is interpolated from parameters held at corner nodes (vertex points), at refinement (*a*) it is a function of parameters at nodes 1–6, and at refinement (*b*) it has additional parameters for each new node, such that the field description along the boundary between the two original elements is a function of parameters at nodes 2, x and 5. If element E1 had not been refined at state (*b*), x would be treated as a *hanging node*, meaning its parameters are not stored, but calculated in terms of the parameters at nodes 2 and 5 to maintain continuity between the two elements.

It is important that the hierarchical relationships between these levels of mesh refinement are maintained, in particular the mappings of element chart coordinates. This supports cases where more than one field will be represented by different patterns and levels of refinement, and also post-processing of a full time-varying simulation. The development of this type of hierarchical mesh refinement in FieldML is likely to exploit the treatment of parameters as fields, to map parameters of refined meshes back to coarse mesh parameters and handle cases such as hanging nodes.

**Figure 6:**
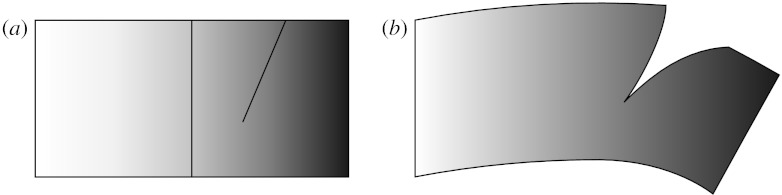
Connectivity of coordinate field versus material property based on meshes from figure 5. (*a*) Shaded material property shown on coarse mesh with cut line shown for reference and (*b*) deformed geometry including cut, defined on highly refined mesh.

Figure 6 shows the situation of two fields defined over different mesh refinements from figure 5. The shading indicates the variation of a material property such as stiffness, which can be described throughout a simulation on the original, unrefined mesh, even if the coordinate field is refined several times to accurately describe a detailed deformation involving a cut in the mesh. This shows that one has to be careful when talking about connectivity; it is not a universal property of a hierarchical mesh, but a property of a field at the particular mesh refinement it is defined over at any instant.

### (g) Field and domain combinations

Fields defined in different FieldML models are not compatible with each other and mathematical combinations of them are not permitted. To work around this restriction and reuse objects from other models, the use of the special *import* field type is required.

Fields are not permitted to depend, even indirectly, on themselves. Being non-cyclic (non-recursive) is a requirement for a declarative language such as FieldML.

Within a FieldML model, certain rules of field and domain combination apply. A field created using an operator acting on two or more fields defined over different domains will be defined over the intersection of those domains. Rules and language constructs to govern or enable operations on fields defined over unrelated domains are still under discussion. An example is multiplying a spatially varying field by a time-varying field with the intention of having the result defined over space and time. Domain compatibility may be a requirement for certain field operators in the same manner as units and value types. Note that the current thinking on model-wide constants and simple variables is that they may be combined in operations with any other field.

## 4. Example: finite-element interpolation

This section shows a simple mock-up of a FieldML data file to illustrate how complex field descriptions can be constructed from simple building blocks. Be aware that it is neither an existing format nor is it ready to be proposed as a standard, and where it is not clear the accompanying comments should clarify the intended meaning. It is written in extensible markup language (XML) format (www.w3.org/XML) since it is understood by many people and is a strong candidate for use in the eventual standard.

The example defines a two-dimensional coordinate over the mesh in figure 5*a*. It consists of only a single model, hence all items within the model tag denote named fields. Units are not used in this example and XML header information is omitted.

The key part of the example is the definition of a two-dimensional coordinate field using the following interpolation functions:

$$y_{i}=\sum_{j=1}^{4}\varphi_{j}a_{ji},$$

with bilinear basis functions *φ*_*j*_ expressed in terms of the element coordinate system components *x*_1_ and *x*_2_,

$$\varphi_{1}=(1-x_{1})(1-x_{2}),$$

$$\varphi_{2}=x_{1}(1-x_{2}),$$

$$\varphi_{3}=(1-x_{1})x_{2},$$

$$\varphi_{4}=x_{1}x_{2}$$

and given element field parameters *a*_*ji*_.
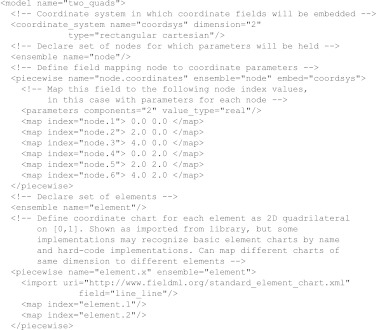

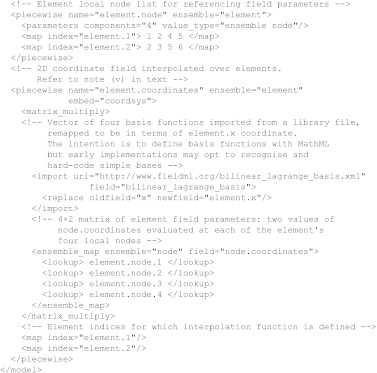


Additional notes are as follows:

- The node ensemble is declared but the objects making it up are not declared until used in the piecewise field ‘node.coordinates’.
- Domain name prefixes are used to distinguish the names of parameter field node.coordinates defined over the node domain, and the continuous coordinate field ‘element.coordinates’ defined over the elements. It is under consideration that this be extended to formally define fields within the scope of parent fields. Both of these fields are embedded in the coordinate system domain ‘coordsys’, indicating to the software how to interpret or transform their values.
- The ‘element’ ensemble domain gains only two-dimensional element charts on definition of the piecewise domain field ‘element.x’. It is still under debate whether a tighter coupling between the concepts making up a FieldML mesh is needed.
- The ‘element.node’ parameter field takes an element reference and returns a value consisting of a list of four references to objects in the node ensemble.
- The key part of the example is the definition of the interpolated field element.coordinates, according to the equation above. In this example, the basis functions are assumed to be imported from a library and mapped to be in terms of the element coordinate system field element.x. The element field parameters are evaluated as the values of the node.coordinates field at each of the local nodes listed for the element.node field. This is the standard finite-element field function, which is linearly dependent on parameters, and only the basis function depends on locations in the element chart. Note that any general function of element.x and other fields is possible here, including countless mappings of element field parameters.

## 5. FieldML implementations

FieldML concepts are currently being implemented in two open source software projects, both released under business friendly licenses.

**Figure 7:**
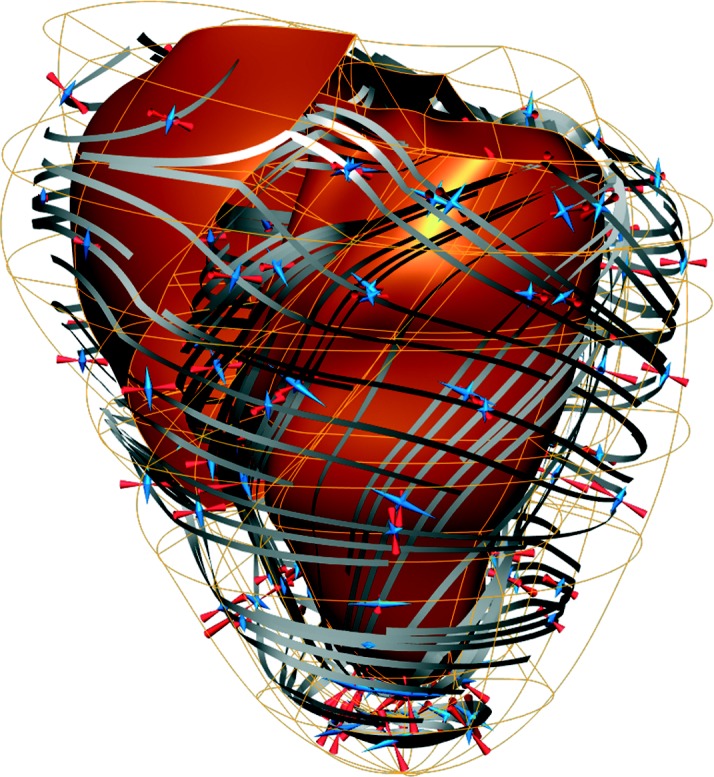
Visualization of a deforming heart in Cmgui, from a simulation performed using CMISS (see Nash & Hunter 2001). Here the coordinate field is defined in a prolate spheroidal coordinate system, and interpolated over finite elements using different basis functions for each component. Streamlines show the muscle fibre coordinate system with respect to which material properties for the simulation were defined; it is defined by interpolating Euler angles over elements, which transform an orthonormal coordinate system relative to an initial orientation aligned to the gradients of the coordinate field with respect to the element chart coordinates. Displacement gradient operators applied to the coordinate field at various simulation times and relative to the initial state are further converted into large strains; eigenvalues and eigenvectors of the resulting matrix give the principal strains, which are visualized as arrows, blue and outward pointing for extension, red and inward pointing for compression.

The first is Cmgui (www.cmiss.org/cmgui), the field visualization component of the Auckland Bioengineering Institute's CMISS software platform, now developed in collaboration with other sites. Cmgui is a powerful visualization package offering high-quality graphics, complex field representations with a large number of field operators and manipulation tools, and is being extended to solve optimization problems expressed via field operators. Defining new fields via operators has proved itself as an extremely modular approach to software development: new operators are easy to add, and using them in clever combinations routinely solves complex representation and visualization problems with no extra coding, as demonstrated in figure 7 (see also Christie *et al*. 2002). The core functionality of Cmgui is being exposed as an API enabling use by other software and scripting languages, and can be embedded in web pages via its Zinc component.

Cmgui does not yet implement all parts of the FieldML proposal in this paper. Most notably, it cannot describe fields with the generality offered by the ensemble and piecewise operators described in §§3*b* and 4, and is limited to fixed element chart shapes and a fixed, albeit rich, set of basis function types. However, it supports image-based fields, image processing and other field operators, which may remain extensions to any FieldML standard. Its implementation is oriented towards interactive visualization that has the requirements for propagating field change messages to automatically update graphics and other coupled features, which is not necessarily needed in other applications. Complexity issues in field visualization include handling time variation, possibly with mesh refinement, and user demands to visualize every aspect of a model.

The second implementation is openCMISS (http://sourceforge.net/projects/opencmiss), which is a computation engine designed to solve very large problems in bioengineering and other arenas using finite-element, boundary-element and other methods. Its implementation is oriented towards parallel computing environments, notably distributed memory systems coordinated via the Message Passing Interface (MPI) standard. openCMISS is also being developed collaboratively between multiple organizations, and similar to Cmgui it partially implements the FieldML ideas expressed in this paper. Particular complexity issues for distributed parallel computation include mesh and field partitioning to solve parts of problems on separate computation nodes, and the need to map solution field parameters to a common distributed vector.

Current developments focus on building a common API for applications working with FieldML models. This will be based in part on the Cmgui API. The API will allow the client software to create and manipulate fields in the chosen implementation and to serialize fields into and out of various data stores. It would also act as an interface between implementations, enabling, for example, a very large field to be stored on a compute cluster using openCMISS, yet visualized from Cmgui on a remote workstation.

Library implementations of the API should be able to translate other data formats to and from FieldML constructs. There is also a need to develop one or more native FieldML serialization formats, one of which should be a text-based file format useful for testing, learning and small problems, a likely candidate format being XML. Larger problems will require fast binary storage and retrieval in parallel; at this time, a likely candidate for implementing this is the Hierarchical Data Format, HDF5 (www.hdfgroup.com/HDF5). Finally, to support multiple FieldML implementations and applications with their own data structures, the FieldML library should be able to act as a translation layer with partial buffering of field data as an alternative to a full in-memory representation.

## 6. Discussion

This paper has been written to communicate our current thinking on FieldML in the hope of inspiring debate and participation from the broader modelling community towards the ongoing development of a powerful standard for modelling and interchanging fields.

The prime objective of FieldML is to ease the task of representing and interchanging advanced computational field representations in software. FieldML defines modular field operators acting on argument fields of particular value types, but which usually do not know the ultimate domain over which those fields are defined. FieldML calls the result of these operators a field, but leaves its domain and other features to be discovered at run-time. This flexible definition maximizes code reuse, since the operators can be applied to both spatially varying fields and fields defined on discrete objects. The result is more powerful field representations from less complex software and computation environments where extra complexity can come by defining additional fields, rather than custom data structures that are difficult to integrate completely.

The FieldML proposal is far from complete. There is much work to be done in all areas including: defining operators; rules; units; field value-type specifications; representing hierarchical meshes and fields; attributes and metadata to convey additional meaning. Additional concepts and complexity may eventually need to be added to the language, but this will only be done if it becomes clear that existing concepts and metadata cannot effectively express the required information or meaning. Even naming many of the major elements such as model (or region) is an important task.

Contributions are also requested to develop the software libraries and implementations of FieldML, which are currently several steps behind the proposals in this paper. Performance optimization is an area of particular importance, with potential for high-level FieldML models to be converted into code optimized for current and future hardware architectures, be they distributed or shared memory systems, with multi-core general-purpose processors or special purpose co-processing units or some other configuration. Inputs on requirements and priorities for software development are invited.

Questions and ideas about FieldML as well as general queries about software implementations can be posted on the ‘FieldML specification’ section of the Physiome Project tracker (http://tracker.physiomeproject.org). There is also a FieldML website (www.fieldml.org) with some additional resources.

An important aspect to be resolved is the integration of CellML and FieldML. There are ongoing efforts to integrate CellML-related tools into the FieldML implementations of §5, but an intriguing idea is that in the long term the two languages could merge. FieldML already has the basic idea of a lumped parameter or variable, and its models are similar in concept to CellML models and components (www.cellml.org/specifications). Even though FieldML serialization formats are in development, CellML and other formats will remain relevant for serializing restricted types of field data. For the time being, it is anticipated that FieldML will leverage off many of the useful ideas from CellML in the areas of units, MathML, code generation and the lessons learned in creating a standard.
